# The association between Type-D personality and in vitro fertilization success among women with unexplained infertility

**DOI:** 10.12669/pjms.41.3.9966

**Published:** 2025-03

**Authors:** Esra Nur Tola, Umran Kilıncdemir Turgut

**Affiliations:** 1 Esra Nur Tola Department of Obstetrics and Gynecology, Istanbul Medipol University Faculty of Medicine, Medipol Pendik Hospital, Istanbul, Turkey; 2 Umran Kilıncdemir Turgut Department of Obstetrics and Gynaecology-Perinatology, University of Health Sciences, Adana City Training, Research Center, Adana, Turkey

**Keywords:** Depression, In vitro fertilization, Type-D personality

## Abstract

**Objective:**

To evalute the association between Type-D personality and in vitro fertilization (IVF) success in unexplained infertile women.

**Methods:**

This was a cross-sectional study conducted at the IVF unit of a tertiary center between September 2017 - December 2019. The 21 items Beck Depression Inventory (BDI-21) and 14 items Type-D Scale (DS14) were noted from 256 unexplained infertile women undergoing an IVF cycle.

**Results:**

Paternal educational level and clinical pregnancy were lower and depression was more prevalent in Type-Ds than non-Type-Ds. There was a positive correlation between BDI-21 scores and duration of infertility and cycle count. Age, paternal educational level, anti-mullerian hormone and metaphaseII number were lower and Type-D personality was more prevalent in women who failed to become pregnant than women who achieved pregnancy. After adjustment of factors affect the IVF success, clinical pregnancy was negatively associated with Type-D personality, age and low paternal educational level, whereas it was positively associated with infertility duration and single embryo transfer.

**Conclusion:**

It seems that having a Type-D personality may negatively affect clinical pregnancy after IVF treatment in couples with unexplained infertility.

## INTRODUCTION

In vitro fertilization (IVF) is potential source of stress and emotional problems due to the treatment itself, the anticipation of pregnancy, and the fear of failure of the treatment.^1^ In recent years, studies have focused on the psychological health of infertile couples. The relationships between infertility and lack of self-confidence, feeling of grievance, guilt and disappointment have been explored.^2^,^3^ It has been found infertile women who have social support, positive personal characteristics, and a satisfactory life with their partners exhibit lower depression.^4^

Type-D personality is defined as a combination of two stable personality characteristics which consists of negative affectivity (NA) and social inhibition (SI). Individuals with having a Type-D personality have a vulnerability to experience negative emotions against situations (NA) and avoid social interactions due to fear of rejection or disapproval (SI).^5^ Type-D personality leads to various diseases, such as diabetes, cardiovascular disease (CVD) and neurological diseases.^6^ It is also thought to be a novel risk factor for increased adverse clinical outcomes of various disorders, including CVD, diabetes, and ankylosing spondylitis^5^,^7^ However, the role of the Type-D personality on IVF success has not been studied. Due to the controversies related to the role of psychological stress on IVF outcome, we aimed to analyze the association of Type-D personality and IVF outcome in unexplained infertile women.

## METHODS

This was a cross-sectional study conducted at the IVF unit of a tertiary center between September 2017 - December 2019. A consecutive series of 256 unexplained infertile women undergoing an IVF cycle were included for this study. Unexplained infertility was diagnosed after normal standard infertility evaluation according to the guideline of the Practice Committee of the American Society for Reproductive Medicine.

### Ethical Approval:

The study was approved by the institutional Ethics Committee (Ref.No: 2012-KAEK-38, Date: August 23, 2027) with the protocol number: 72867572.050.01-157922.

### Exclusion criteria:

Those having a history of any psychiatric disorder (depression, anxiety disorders, etc), chronic medication use, major medical conditions, refusal to participate, or inability to read and understand the Turkish language were excluded. The participants completed the 14-item Type-D Scale (DS-14) and 21-item Beck Depression Inventory (BDI-21) at the beginning of IVF treatment and related scores were recorded.

The validated DS14 scale was applied for the assessment of Type-D personality.^8^ It consists of two subscales with 14 items, each of which is rated from 0 to 4. One of the subscales is NA, and the other is SI. Patients who score high on both subscales (cut-off score ≥10) are considered to have a Type-D personality.

The validated BDI-21 scale -consisting of 21-items - is useful for evaluating the presence and severity of depression.^9^ A cut-off value of 17 or more was established as ‘depression’ and higher scores as indicating greater depression. Mild depression was defined as total standard scores of 17-20, moderate as 21-30 and severe as more than 30.

Controlled ovarian stimulation was performed with short antagonist protocol. Human chorionic gonadotropin (hCG) was administered for ovulation trigger. Fertilization was evaluated as the presence of two pronuclei. Embryos were classified according to their morphology: Grade1 (good quality), Grade2 (moderate quality), and Grade3 (poor quality).^10^ Clinical pregnancy was defined as the presence of fetal heartbeat five weeks after embryo transfer.

The participants were evaluated in two subgroups based on their characteristics. In the first classification, the participants were divided into two subgroups based on the presence of TypeD personality or not and the variables were compared. In the second step, participants were classified according to they became pregnant or not after IVF treatment.

### Statistical analysis:

It were performed with SPSS version 20. For all tests significance level was set at p <0.05. Continuous variables were compared using Student’s t test and Mann-Whitney U test. Chi-Square test were used for the comparison of categorical variables. Correlation analysis was used to determine the relationships between continuous variables. Logistic regression analysis was performed to clarify the impact of Type-D personality on clinical pregnancy.

## RESULTS

The mean age of the participants was 30.8 ± 5.5 years. About 92.2% of participants had primary infertility, and 7.8% of participants had secondary infertility.

### The comparison of Type-D and non-Type-D groups:

Age, BMI, maternal and paternal habits (smoking and alcohol), maternal education, duration of infertility, infertility type (primer or secondary), cycle count, antimullerian hormone (AMH) levels were distributed homogeneously between Type-Ds and non-Type-Ds. Paternal educational level tended to be higher in non-Type-Ds compared to Type-Ds (p=0.04). BDI-21 score was higher in Type-Ds compared to non-Type-Ds (p<0.0001). The findings showed that 5.5% of the non-Type-D group participants reported that they were depressed, while 29% of the Type-D group participants were depressed. Depression was more prevalent in Type-Ds than in non-Type-Ds (p<0.001). However, depression severity was distributed homogeneously between the two groups (p=0.2) (Table-I).

**Table-I:** Baseline characteristics and treatment variables between Type-Ds and nonType-Ds.

	nonType-Ds (n=163)	Type-Ds (n=93)	p value
Age (years)	31.11 ± 5.81	30.49 ± 5.2	0.3
BMI (kg/m^2^)	25.35 ± 4.58	25.85 ± 4.92	0.4
***Paternal education (n, %)***			0.04
Primary	32/163 (19.6%)	30/93 (32.3%)	
Lycee	54/163 (33.1%)	31/93 (33.3%)	
University	77/163 (47.2%)	32/93 (34.4%)	
Infertility duration (years)	5.63 ± 4.08	6.17 ± 4.21	0.3
Cycle count (n)	1.51 ± 0.93	1.53 ± 1.37	0.8
BDI-21 score (points)	6.54 ± 5.91	13.25 ± 7.98	<0.0001
***Depression present (n, %)***	9/163 (5.5%)	27/93 (29%)	<0.0001
Depression severity			0.2
Mild depression (n, %)	4/9 (44.4%)	11/27 (40.7)	
Moderate depression (n, %)	2/9 (22.2%	13/27 (48.1%)	
Severe depression (n, %)	3/9 (33.3%)	3/27 (11.1%)	
Metaphase II oocyte (n)	7.01 ± 4.84	7.43 ± 5.12	0.5
Fertilized oocyte (n)	4.22 ± 3.51	4.53 ± 3.90	0.5
***Embryo quality (n, %)***			0.3
Grade 1	116/133 (87.2%)	66/76 (86.8%	
Grade 2	14/133 (10.5%)	10/76 (13.2%)	
Grade 3	3/133 (2.3%)	0	
***Embryo transfer day (n, %)***			0.06
Day 3	56/133 (42.1%)	36/76 (61.9%)	
Day 4	34/133 (25.6%)	10/76 (13.1%)	
Day 5	43/133 (32.3%)	19/76 (25%)	
Single embryo transfer (n, %) Double embryo transfer (n, %)	106/133 (79.7%) 27/133 (20.3)	55/76 (72.4%) 21/76 (27.6%)	0.2
Clinical pregnancy (n, %)	41/133 (30.8%)	13/76 (17.1%)	0.03

BMI: Body mass index; BDI-21: Beck depression scale; COS: Controlled ovarian hyperstimulation.

Treatment variables (total dose of gonadotropin used, the mean number of gonadotropin administration days, oocyte retrieval parameters, embryo transfer (ET) day, and transferred embryo number were similar between Type-Ds and non-Type-Ds. Regarding IVF outcome parameters, the number of fertilized oocytes, fertilization rate, and the embryo quality were also similar between Type-Ds and non-Type-Ds. Clinical pregnancy rate was more prevalent in non-Type-Ds (30.8%) than Type-Ds (17.1%, p=0.03) (Table-I).

When we evaluated the correlation between BDI-21, NA, SI, and DS14 scores and demographic, hormonal and oocyte retrieval parameters, there was a positive correlation between BDI-21 scores and duration of infertility (p=0.02, r= 0.1) and cycle count (p=0.01, r=0.1). NA score was positively correlated with BMI (p=0.02, r=0.1) and duration of infertility (p=0.04, r=0.1). DS14 score was also positively correlated with BMI (p=0.04, r=0.1). There was no correlation between the BDI-21, NA, SI and DS-14 scores and age, hormone levels and treatment variables (gonadotropin administration day, total gonadotropin dose, fertilization rate, oocyte retrieval parameters, and embryo number) (Data not shown).

### The comparison of pregnant and nonpregnant groups:

Embryo(s) could not be transferred in 47 (18.4%) cases due to fertilization failure (55.3%), poor embryo quality (14.9%), endometrial factor (4.3%), the development of hyperstimulation syndrome (21.3%) and catheter (4.3%). Therefore, clinical pregnancy was evaluated among 209 cases and 25.8% of participants achieved pregnancy. Maternal and paternal age was significantly lower in nonconceived women than conceived women (p=0.004 and 0.008, respectively). There was no significant difference between BMI and habits of couples, and maternal education, infertility type, infertility duration, and cycle count between pregnant and nonpregnant groups. Interestingly, paternal education level was higher in the pregnant group than in the nonpregnant group (p=0.04). AMH was significantly lower in the nonpregnant group than the pregnant group (p=0.04) (Table-II).

**Table-II:** Baseline characteristics and treatment variables of nonpregnants and pregnants.

	Non-pregnant (n=155)	Pregnant (n=54)	p value
Age (years)	31.36 ± 5.75	28.87 ± 4.51	0.004
Paternal age (years)	34.85 ±6.17	32.42 ± 4.29	0.008
***Paternal education (n, %)***			
Primary	38/155 (24.5%)	13/54 (24.1%)	0.04
Lycee	59/155 (38.1%)	12/54 (22.2%)	
University	58/155 (37.4%)	29/54 (53.7%)	
AMH (ng/ml)	2.61 ± 2.15	3.88 ± 3.53	0.04
***Depression present (n, %)***	23/155 (14.8%)	6/54 (11.1%)	0.6
Depression severity (n, %)			
Mild	9/23 (39.1%)	1/6 (16.7%)	0.3
Moderate	11/23 (47.8%)	3/6 (50%)	
Severe	3/23 (13%)	2/6 (33.3%)	
NA present (n, %)	91/155 (58.7%)	26/54 (48.1%)	0.2
SI present (n, %)	78/155 (50.3%)	27/54 (50%)	1
Type-D present (n, %)	63/155 (40.6%)	13/54 (24.1%)	0.03
Total gonadotropin dose (IU)	2340.29 ± 1047.16	3447.5 ± 413.06	0.5
COS duration	9.85 ± 1.29	10.33 ± 2.3	0.6
MII (n)	7.09 ± 4.38	8.88 ± 5.16	0.01
Fertilized oocyte (n)	4.65 ± 3.38	5.16 ± 3.71	0.3
Day 3 embryo transfer (n, %) Day 4 embryo transfer (n, %) Day 5 embryo transfer (n, %)	81/155 (52.2) 28/155 (18.1%) 49/155 (29.6%)	22/54 (40.8%) 16/54 (29.6%) 16/54 (29.6%)	0.1
Single embryo transfer (n, %) Double embryo transfer (n, %)	121/155 (78.1%) 34/155 (21.9%)	40/54 (74.1%) 14/54 (25.9%)	0.5
***Embryo quality***			
Grade 1 (n, %)	132/155 (85.2%)	5/54 (92.6%)	0.3
Grade 2 (n, %)	20/155 (12.9%)	4/54 (7.4%)	
Grade 3 (n, %)	3/155 (1.9%)	0	

AMH: Antimullerian hormone; NA: Negative affectivity; SI: Social inhibition. COS: Controlled ovarian hyperstimulation; MII: Methaphase II; ET; Embryo transfer.

**Table-III:** The association between Type D personality, depression and clinical pregnancy.

	B	p value	OR	95% CI for OR	

				Lower	Upper
Paternal education (university)		0.008			
Paternal education (primary)	-0.9	0.06	0.4	0.15	1.05
Paternal education (Lycee)	-1.36	0.002	0.25	0.1	0.61
Infertility duration	0.13	0.01	1.14	1.02	1.26
Single embryo transfer	1.08	0.01	0.33	0.13	0.82
Type D personality	-0.8	0.024	2.38	1.11	5.06
Maternal age	-0.19	<0.0001	0.82	0.74	0.9
Constant	4.96	0.002	143.18		

Covariates: Maternal age, paternal age, Depression present, BMI, maternal education, paternal education, infertility duration, induction protocol, COC, MII, embryo transfer day, embryo quality, transferred embryo number, type of infertility (primary/ secondary), previous IVF cycles.

There were no differences between the conceiving and non-conceiving women concerning BDI-21, NA, and SI scores (p=0.05). About 13.9% of the participants reported that they were depressed, while approximately 34.5% had mild depression, 48.3% were moderately depressed and approximately 17.2% had severe depression. Depression and depression severity were distributed homogeneously between pregnant and nonpregnant groups. Type-D personality was more prevalent in women who failed to become pregnant than women who achieved pregnancy (p=0.03) (Table-II). There were no statistical differences regarding the total gonadotropin dose and administration days, poor ovarian response, ovarian hyperstimulation syndrome, the number of fertilized oocytes, ET day, transferred embryo number and embryo quality between the conceiving and non-conceiving infertile women. Total retrieved oocyte and metaphaseII numbers were significantly higher in the pregnant group than in the nonpregnant group (p= 0.03 and 0.01, respectively) (Table-II).

We evaluated the association between Type-D personality and clinical pregnancy using multivariate logistic regression. Variables which are known to affect IVF success and were different between pregnant and nonpregnant groups and between Type-D and non-Type-D groups, and also the variables which were correlated with BDI-21, NA and SI scores (infertility duration, maternal age, paternal age, level of education, BMI, induction protocol, COC, MII, embryo transfer day, embryo quality, transferred embryo number, type of infertility [primary/ secondary], and previous IVF cycles) were used as covariates to minimize the risk of failure. After adjustment of the abovementioned variables, we observed that clinical pregnancy was negatively associated with Type-D personality (p=0.02), maternal age (p<0.0001) and low paternal educational level (p=0.008), whereas it was positively associated with infertility duration (p=0.01) and single embryo transfer (p=0.01).

## DISCUSSION

Several biological factors have been reported to affect IVF success, however, psychological state and personality type could be significant for IVF outcome and interact with the biological factors. To our knowledge, this study is the first to focus on the role of enduring personality traits and depression on IVF success. We found a close relation between Type-D personality and poor IVF success, defined as clinical pregnancy in unexplained infertile women even after adjusting factors that are known to affect IVF success.

Several studies have evaluated the effects of anxiety and depression on IVF success. Psychological wellbeing has been demonstrated to increase the success rate of IVF.^11^,^12^ Linsten et al. found no effect of depression and anxiety on IVF cycle cancellation rates and pregnancy rates among women with different infertility etiology.^1^ Li et al. found that 10% of patients experienced baseline psychological stress (both anxiety and depression) among tubal infertile women. They concluded that baseline stress could negatively affect the clinical pregnancy rate in women with tubal infertility.^13^ No association was found between depression and clinical pregnancy rate in women with different infertility etiology.^14^ In our study, 13.9% of participants reported they were depressed, and the depression rate and severity were similar between women who conceived and those who could not conceive. Evaluating only women with unexplained infertility could be a reason for the relatively increased depression in our study population since knowing what causes infertility can relieve the woman psychologically. Evaluating emotional state with different scales and in women with different infertility etiology could be a reason for the discrepancy between the results in previous studies.

To our knowledge, no study has evaluated the role of personality type on IVF outcome in the literature. Previously, we found a close relation between Type-D personality and infertility.^15^ Csemiczky et al.^16^ evaluated the personality profiles measured by the Karolinska Scales of Personality (KSP) and demonstrated that higher scores on suspicion, guilt and hostility in infertile women than fertile ones. Berg et al.^17^ described an ‘infertility personality profile’ characterized by feelings of frustration, anger, depression and guilt, which were observed as a response to being infertile. We found lower clinical pregnancy rates in women with Type-D personality compared to non-Type-Ds. Also, after the adjustment of variables that are known to affect IVF success and Type-D personality, the negative association between Type-D personality and clinical pregnancy rate persisted. Increased pro-inflammatory immune activation, oxidative stress and cortisol levels in cardiac patients was found to be related to Type-D personality.^18^

Psychological situations may also cause alterations in endocrine and sympathetic nervous systems.^19^ Biological pathways, particularly oxidative stress and immune activation, increased stress hormones and other biomarkers could support the biological plausibility of Type-D as a risk factor for poor IVF success. The relatively small sample size may be responsible for the statistical significance regarding lower clinical pregnancy rates in Type-Ds. Socio-demographic characteristics usually affect the psychological situation. Our research has shown some unexpected associations between Type-D personality and socio-demographic characteristics. Interestingly, paternal educational level tended to be significantly higher in the non-Type-D group than the Type-D group. Well-education of the male partner may positively affect the personality type of his wife. Thus, a more social and more positive view of women’s lives may affect the success of IVF.

The findings obtained from this study suggest a correlation between the duration of infertility and being at risk of depression and NA. Increased BMI was also correlated with increased NA and DS14 scores in our study. Infertility duration and age are significant for a woman who perceives fertility as her existential role of participating in the continuity of her family. As time passes, the sense of identity and self-esteem is deeply discrediting in older women.^20^ No correlation was found between the scores of scales and subscales and age, hormone levels, treatment variables, fertilization rate and embryo number. Similarly, Csemiczky et al.^16^ found no association between anxiety and personality scores and hormonal measures. The lack of association between the scores of the scales and basal hormones in our study is an expected result because basal hormone levels are an objective measurement regardless of the patient’s personality or emotional state. The ICSI procedure bypasses many stages. Thus, a similar fertilization rate and embryo number is also an expected result.

Many study have been focused on several biological factors for IVF success, however, psychological factors and personality type have not yet well-studied for IVF outcome. A notable strength of the study lies in being one of the rare studies that sheds light on the impact of Type D personality on IVF success. Another strength is using standardized measures and evaluating only unexplained women due to the group homogenization. Additionally, it is valuable for examining other social parameters influencing Type D personality and detailing these in the regression analysis. The biological pathways and underlying mechanisms that may lead to adverse IVF outcomes in Type-Ds should be investigated in further large sample-sized studies. This study may also paves for further research that evaluate whether psychological assessment of infertile couples as a part of their infertility treatment may improve IVF outcome or not.

### Limitations:

It is the cross-sectional design. A longitudinal study would be able to show associations more definitely. The relatively small sample size is the main limitation of our study.

## CONCLUSION

Having a Type-D personality may negatively affect the success of IVF treatment, especially for targeted patient subgroups.
